# Dental Physiology

**Published:** 1871-07

**Authors:** G. R. Thomas


DENTAL REGISTER.

VOL. XXV.] JULY, 1871. [NO. 7.

### Communications.

### DENTAL PHYSIOLOGY.

BY G. R. THOMAS, D. D. S.

In presenting a single article, upon so *vast* and *boundless* a subject as "Dental Physiology," it certainly can not be expected that its author can even *generalize*, much *less particularize* upon the various points of interest as they daily occur to the dental practitioner, and *especially* to the man who is *allied* to his profession, and is "thoroughly awake to every good work," but only to enter the field at random, and to scatter thoughts in like manner; and if any shall fall upon good ground, and spring up, and bear fruit to the good of the profession and to humanity, the object sought by your humble servant will be achieved. First of all, *however*, a man to be a successful practitioner of dentistry, must needs have not only a thorough knowledge of the organs with which he has to deal, but a sound and reliable judgment upon which to base his operations, without which all his theory will prove about as useless as would a *fine* and *beautifully* constructed watch, perfect in *all* its parts, except its main spring, which had been left out by its maker. He should be a man of *sound body* as well as mind, temperate in all his habits, that his patients may have the full benefit of what justly belongs to them.

The men who build your house, till your ground, or drive your coach, *may* be men who are regular partakers of the flowing bowl, visitors of the gaming table, *or* closely allied to many other vices, and *still* be competent to perform all the duties required of *them*, in order that they may receive the remuneration they demand, and can only *expect* for the labor performed.

It is true the practice of dentistry is one of *real* labor, but is there one among us, *who* after spending a half day in *packing* and *burnishing* gold, would be satisfied in receiving the *same* compensation as does the bricklayer for a like amount of time spent in his vocation, when perhaps it has required no less *time* for *him* to fit himself for *that* branch of masonry, than it has you to acquire the art of packing and burnishing gold? But I trust there are none among us who are willing to accept the somewhat prevalent idea, that the "*ultimatum*" consists in knowing *how* to fill a tooth *well*.

*Nor* are there any among us, who would care to have his patient believe that the $15 or $20 he has received from him for the filling of a tooth, was in consideration of the *actual manual* labor performed; far from it, *because* our labor is no more worth $5 or $10 an hour, than is that of the watchmaker, who sits at his bench eight or ten hours of the day, were it not for the knowledge we have, or are *supposed* to have, of the delicate organs with which we are dealing; and just in proportion to the knowledge we possess of those organs, their structure, their development, the functions they are called upon to perform, the idiosyncrasies of our patients, and various diseases attending them, and the means at our command to *arrest* and *prevent said* diseases, in just such proportion, shall we be enabled to command the confidence and respect *of*, as well as the remuneration for our services from our patients.

What would we say to the dentist who would mallet a beautiful and well constructed filling into a cavity containing an exposed pulp, without first protecting it from the action of the gold? We should at once set him down as an ignoramus, and *he* would doubtless be treated to the exquisite pleasure of having his patient to *sit* down on or about the day following, for the examination of a sick and painful tooth.

He might succeed, however, in making his patient believe for a time, that the trouble was owing to the influence of the mallet. or that the tooth was naturally depraved, for want of a better reason to give. Or what would we say of the man who upon finding an exposed nerve, would at once resort to *destruction* and *extirpation?*

We should say, he belongs to a class, who we hope are fast becoming extinct; or what say you of the man who succeeds in making his patients believe that the tooth which *aches* demands *immediate* extraction, or that the extraction of good teeth is necessary to the securing of an artificial denture, or that salivary calculi is a product of the stomach, —as I have myself heard it said—or that the extraction of six year molars would be followed by second teeth.

Such practice may do for a time with a certain class, but the "*truth* is *mighty* and *will* prevail." But this is not "Dental Physiology." First then a word upon dentition, for when we take into consideration the fact, that one-sixth of children perish from the accidents of dentition,—in which medical statistics will bear me out—it becomes evident that we as *medical* advisers can not give it too much attention. *I* can not believe that dentition of itself is attended with very serious or very painful phenomena. It does not seem possible that nature would attach to the development of any of our parts, real danger to life, or such suffering as must directly threaten it. And yet, experience teaches that dentition is to be dreaded. It is therefore natural that we as practitioners should seek to discern what are the *causes* which can derange dentition, and render it fatal or painful.

Opinions have *varied* upon this important subject. *Some* have seen nothing to cause these accidents, but the degree of *sensibility* which *nature* has imparted to young children. But *such*, I fancy, have not *considered* that the gums are not very sensitive organs, that the nerves do not play a very important part in dentition, and that there *are* in the economy of infancy, *many* acts which much more than the development or formation of the teeth might occasion these serious accidents which are apt to occur at this period.

It is doubtless *true*, that morbid sensibility may contribute greatly to the suffering which infants experience in dentition. *But*, to look no farther than this, would be to examine but a small part of the causes that render this period dangerous. Others have looked no farther than to the fact of the pressure made by the roots of the teeth upon the alveolar periosteum.

*These* have not considered that the *shooting* of the teeth, characterized by the enlargement of the alveolar walls, the distention of the gums, occasioned by the formation of the body or of the crown of the tooth, is an epoch often more dangerous than that of the organization of the roots, which besides would do much more harm in compressing and binding the soft and pulpy part of the tooth, than the serous membrane which performs the function of a periosteum, and lines the interior of the gum, and the proper cavity of the teeth. Others again believe the whole difficulty to result from the resistance of the gum to the passage of the teeth. Now then, if the organic state of the system, if the compression of the periosteum, or if the resistance of the gum, (which *must* be opened by bodies, some of which are blunt it is true.) are the principal sources of *danger* in dentition, why, I ask, are not these dangers *constant* and *inevitable?* Why are not *all* infants exposed to the disorders in question? Why, also, does it happen, that the shooting of the teeth, (which must not be confounded with the eruption,) is often the only period of danger? Why do not brutes partake with *men*, the sorrowful evils of dentition? Why are *second* dentition and the cutting of the wisdom teeth unaccompanied generally with these disorders? And why is the *end* of the period of dentition, ordinarily more critical than the commencement? But it is not my purpose to enter into a a detailed dissertation of this subject of dentition, but simply to throw out a few suggestions. I can not refrain from saying just here, however, that it is my belief that the accidents that surround and mark the epoch of dentition, are almost all foreign to the infant. *For*, if it were not so, but on the contrary, if dentition were a malady, we could none of us expect to enjoy good health until eighteen or twenty years of age, since we are getting teeth the greater part of that time. Although this operation of nature be accompanied by fever, convulsions, and many other troublesome phenomena, yet we find so many cases of exemption, that it would be improper to consider these accidents natural, and to hide the fact that they proceed from too great a fullness, or from the corruption of humors put in motion by the pain of dentition.

It is true that the eruption of the teeth is scarcely ever *unattended* with pain, and indeed a little fever; but I believe if the blood and the humors are neither acrid nor too abundant, these accidents are so inconsiderable that they gradually disappear without further trouble.

And in summing up this whole matter of dentition, I am induced to believe, that when a child has come to its full term, when the mother has exercised prudence during pregnancy, has committed no improprieties in *eating* and *drinking*,—*and by-the-by this means a great deal*,—and the child had good milk from its birth, the teeth are cut easily enough, and with little pain.

And just here I am inclined to think that I can not proceed with this subject of Dental Physiology to better advantage, than to call your attention to what I believe to be the *true causes* of the physiological defects of the teeth of the American people, of this generation. I might go on and tell you of the *formation* of the teeth, of their *composition*, of their *blood vessels*, arteries, veins, nerves, nervous matter, and of all the diseases they are heir to, their treatment, etc., etc., but all these you have laid down in the text books, besides they are nearly always the subjects under consideration when writing upon Dental Physiology. And these, of course, are all necessary considerations; but I believe there is something far beyond these, and that *we*, as honest *advisers*, are bound to look after, and to give our patients the benefits of our researches, if we would make the *teeth* of coming generations, anything more than what we have in the present.

In *one* respect, if no *other*, the teeth differ very materially from other organs of the animal economy. They have no recuperative power, but to compensate for this defect, they are made of materials much more indestructible than those of any other organs; and being properly supplied with the elements necessary for the formation and nourishment, and used in accordance with nature's laws, they last the lifetime of the animal, and are not subject to disease. Is not this plainly proven in the lives of the lower animals? It is true, we oftentimes see decayed teeth in the mouths of the lower animals. *But*, what are the facts in the case? *Never* except they have been injured by accident, or have been made to eat improper food in the service of man. The *cow*, for instance, is a striking illustration of this. I have seen it stated but a short time since from *good authority*, that the teeth of the cow made to live upon the dregs from the breweries decay in a very short time. And, *furthermore*, that the teeth of those that were located a few miles distant from the breweries, lasted much longer than those located close at hand, thereby giving good evidence of what *I have* strongly advocated for some years, to-wit: That sudden changes of temperature is one of the causes of decay. For in this case the cause is clearly more mechanical than physiological, the still feed being given to those near at hand while hot, but becoming cool before being given to those at a distance. To understand this statement, we have but to consider that the *enamel* which protects the dentine from decay, and *glass*, are *very similar; both*, at *least*, requiring the combination of silex with some alkaline principle, and therefore must be alike under the physical law of expansion and contraction. Doubtless, *all* will admit the fact, that if I were to take a very thin and irregularly shaped piece of *glass*, and immerse it in water at a temperature of 200 degrees,—nearly the boiling point,—and then immediately change it to ice water temperature,—subjecting it to such changes for only a short time,—that it would not require a very strong magnifying power to discover an abundance of little checks, small, it is true, yet some of them large enough to admit the fluids of the mouth, and thereby giving the monster, decay, fearful and boundless sway. And is not this the very thing, that we as a people, are constantly doing with our teeth? Exposing them one moment to almost boiling hot tea and coffee, and at another to ice water or ice cream. An almost instantaneous change of from 80 to 100 degrees. And if the enamel once becomes broken in such a manner as to admit the fluids of the mouth, I believe that decay is inevitable, and that at no distant day.

But this is only *one* of the many causes of decay, this being what we may denominate *mechanical*, while the greatest of *all* is purely physiological, which I believe our ancestors for the most part are responsible, for we know that the *universe*, and all things in it, whether of mind or matter, are moved and regulated by fixed laws, and so long as they remain *undisturbed*, all is well, but the violation of any *one* is *sure* to bring its penalties. All *suffering* and all defects may therefore be traced to the violation of *some* law of our being, and the question now comes, what law have we violated, *for* that we—or our ancestors rather—are guilty, is certain, *or* why are not our teeth as good as those of the lower animals. That they are made as perfect if the right materials are furnished, there can be no doubt, for all nature as well as the Bible, teach us that the crowning work of God's creation was man. To whom He says: "Behold, I have given you every herb bearing seed, which is upon the face of the earth, and every tree, in the which is the fruit of a tree yielding seed; to *you* it *shall* be for meat." He also says the life of man shall be "three score years and ten," but the violation of nature's laws has reduced it to an average of thirty-three years.

The child, while dependent upon the mother, gets lime, phosphorus, silex, potash, and all the other elements of which the teeth are composed, in just such proportion as she gets it from the food nature provides, containing these elements in their natural proportion. But where can the child in its forming state, *get* these necessary elements, whose mother lives principally on starch, butter and sugar, neither of which contains a *particle* of *lime*, *potash*, *phosphorus* or *silex?* Nature performs *no* miracles. She makes teeth as glass is made, by combining the elements which compose them according to her own chemical principles. And this illustration is the more forcible, because the composition of the enamel of the teeth and of glass, is very nearly identical—both at *least* requiring the combination of silex with some alkaline principle.

If then, the mother of an unborn or nursing infant, lives on white bread and butter, pastry and confectioneries, which contain no silex, and very little of the *other* elements which compose the teeth, nothing short of a miracle can give her a child with good teeth, and especially with teeth well enameled. And thus is the iniquity of the fathers visited upon the children.

This applies more directly however, to the deciduous teeth, for the second tooth of a child is, for the most part, formed from materials furnished in the blood, secreted or taken up, and used in forming it by a mysterious power imparted to the little gland or nucleus, placed for that purpose directly under the first tooth, but entirely independent of it. And this mysterious power acts in this, as in all other organs, with unering certainty, and if the right elements are furnished in the blood, will be sure to find them, and furnish them in right proportions to the forming tooth.

But the trouble is the child's appetite is early educated to the use of unnatural food, by which these diseases and sufferings are so early commenced, and of course will follow on thoughtlessly, in the way in which its parents have started it, till it inevitably falls a pray to the diseases which are thus induced and perpetuated. I am often asked when discoursing upon this subject to my patients, "What articles of food *ought* we to eat, in order to make good teeth?" I answer, everything that grows will make good teeth, if eaten in their natural state, no elements being taken out, for every one of them does make good teeth for horses, cows, sheep, and all other animals that live on natures productions, pure and unadulterated. But when preaching this doctrine of diet, I am often asked, "Don't you think poor teeth are hereditary?" It is true some of the phenomena of hereditary diseases are very mysterious and very curious, but I believe that what we inherit in the teeth, is merely the want of the necessary elements, and in that way having its influence upon posterity.

I have often heard a mother console herself for the defect of her childs teeth, by saying, "*Well*, my teeth are bad, and my mothers were, and therefore, poor teeth are inevitable in my family." The fact is, such a mother and *grandmother* have lived on carbonaceous food, and drank hot *tea* and *coffee*, and the consequences *were* inevitable. But I am thoroughly convinced that if such a mother will commence to feed her child upon natural food, while it is young, and teach it to *continue* it in its older years, and avoid other causes of defective teeth, the consequences will be *also* inevitable, and the next *generation* will begin with better teeth, and by continuing the *course*, will continue to grow better, transmitting them from generation to generation. Let us then, in the name of Him whose we are, and to whom we expect to give an account "of our stewardship," strive not only to alleviate the sufferings of those around us now, but seek to educate them into such habits of life, as shall lead to a better and stronger physical organization, and as a natural consequence, good teeth will follow. Thus shall the members of our honored profession reap as their reward the respect and love of a grateful people.

